# Advancing a taxonomy of proxemics for socially aware robot navigation 

**DOI:** 10.3389/frobt.2026.1800762

**Published:** 2026-05-20

**Authors:** Ehud Nahum, Yael Edan, Tal Oron-Gilad

**Affiliations:** Department of Industrial Engineering and Management and Agricultural, Biological, Cognitive Robotics Initiative, Ben-Gurion University of the Negev, Beer-Sheva, Israel

**Keywords:** human-aware navigation, human-robot interaction, proxemics, socially aware navigation, social-robot navigation, taxonomy

## Abstract

Socially aware robot navigation requires robots to move among people in ways that respect human social norms, comfort, and perceived safety. Proxemics, the regulation of interpersonal space, plays a central role in this process. Applied HRI work often relies on simplified, static representations of personal space, overlooking the dynamic, asymmetric, and context-dependent nature of proxemic behavior observed in real-world interactions. The literature reflects a clear progression from simplified, concentric representations of proxemics toward increasingly context-sensitive and interaction-dependent models. This evolution indicates a growing consensus that interpersonal comfort cannot be adequately captured by a single, universal geometric shape. Instead, proxemic representations vary as a function of interaction context, task demands, cultural norms, and environmental constraints. To build on this evolution, we propose a comprehensive taxonomy of proxemics for socially aware robot navigation addressing gaps in the literature. Grounded in an extensive review of proxemics-related HRI studies published between 2020 and 2025, the taxonomy was developed through a hybrid methodology that integrates a top-down analysis of established HRI taxonomies and an AI exploratory approach with a bottom-up extraction of variables from 39 empirical studies. The resulting taxonomy systematically organizes proxemic dimensions into four interrelated clusters: Human, Robot, Environment, and Context. Together, these clusters capture the key variables shaping proxemic form (shape geometry and the scale of the personal zone boundary) and dynamics, including human activity and posture, robot design and behavior, environmental structure, task context, and the dynamic spatial properties of proxemics as captured by their metrics (the proxemics output variables). The proposed structured taxonomy of proxemics will inform the design of socially adaptive robot navigation systems and provide a foundation for future empirical research. Our analyses reveal significant gaps in current research practices, including limited consideration of interactions among multiple variables, overreliance on static laboratory settings, and insufficient integration of contextual and human-centered variables. To address these limitations, we propose future directions.

## 1 Introduction

Social robots must navigate spaces shared with humans in ways that are both socially acceptable and safe (Gao and Huang, 2022). Socially aware navigation thus rests on a robot’s ability to perceive and respect social conventions, particularly those related to personal space, to maintain comfortable interactions with humans while achieving its navigation goals (Torta et al., 2012). Therefore, effective navigation in diverse physical and social environments requires robots to model human behavior patterns, preferences, and perceptions of shared space, and to interpret and adapt to human social rules and behaviors (Belhassein et al., 2022; Leichtmann and Nitsch, 2022; Kim S. et al., 2024). One influential concept informing such behavior is proxemics, which studies how humans perceive, manage, and regulate interpersonal space (Walters et al., 2009). Originally developed in psychology to study human-human interactions, proxemics examines how individuals perceive, regulate and manage social encounters in physical space (Hall, 1966). This concept has been extended to HRI, where it highlights the need for aligning robots’ navigation behavior with humans’ expectations of personal space. Within this context, several studies define “personal distance” as the minimum spatial separation required between humans and robots, while considering spatial and temporal factors (Kim S. et al., 2024; Nolte et al., 2025).

Recent surveys of socially aware robot navigation address many key aspects of this domain, but they often overlook essential elements, such as the type of robot and the influence of robot-specific characteristics on navigation strategies (Singamaneni et al., 2024). As a result, there is a growing recognition that *user-adaptive robot behavior* requires more sophisticated human-aware capabilities on the part of the robot. These capabilities include incorporating robot-specific parameters, such as embodiment, size, and mobility and exploring alternatives to traditional proxemics, which are largely based on human-human interaction metrics. Moreover, multiple interacting variables that affect user comfort in HRI must be considered systematically when developing a robot social navigation model (Rios-Martinez et al., 2015; Samarakoon et al., 2022b). In particular, Gérin-Lajoie et al. (2008) highlight that many proxemic challenges arise from an incomplete understanding of human spatial behavior, raising fundamental questions about how personal space should be modeled, and how robots can effectively represent and reason about this space.

Against the background of these limitations, we propose a taxonomy centered on proxemics that addresses key gaps in existing frameworks for socially aware robot navigation. Unlike prior HRI classifications that focus primarily on high-level proxemic concepts or behavioral strategies, our proxemics taxonomy systematically integrates robot and human characteristics with environmental and contextual variables, thereby defining the output variables of the proxemics spatial metrics (shape, measurement method, and morphing). In so doing, it enables more consistent comparison and standardization across studies, while providing a structured foundation to guide future research and the practical deployment of proxemics for socially adaptive robots.

This paper is organized as follows. Section 2 reviews the relevant literature, introducing the concept of proxemic shapes and their role in HRI. Section 3 details the methodology used to develop the proposed proxemics taxonomy. Section 4 defines the gaps between HRI theoretical perceptions and variables extracted from existing proxemics-related studies. Section 5 presents the proposed integrated taxonomy and its core components. Section 6 discusses the implications of the proposed taxonomy, examines limitations in existing research, and highlights key challenges and opportunities for future work.

## 2 Related work

### 2.1 Definitions of proxemics shape in HRI

People perceive and regulate the space and distance they maintain from others in ways that are shaped by culturally specific norms and situations (Brown, 2006). Personal space is thus a central concept in proxemics, as humans subconsciously account for the personal space of others during social interaction and while navigating the environment (Liu et al., 2021). The study of interpersonal proxemics was formalized by Hall (1966), who defined four concentric, circular regions surrounding an individual to characterize different interaction and communication distances. Several works relating to HRI proxemics have adopted Hall’s circular representation of personal space (Kirks et al., 2020; Repiso et al., 2022; Singh et al., 2023; Nguyen and Ngo, 2024). Similarly, Hecht et al. (2019) modeled personal space as circular and distinguished zones based on interaction type. However, other studies have challenged this assumption, providing evidence that personal space is not necessarily circular and may exhibit more complex shapes (Rios-Martinez et al., 2015).

One of the first reviews of attempts to define a proxemic space as a zone that changes shape and size with human behavior and context (Rios-Martinez et al., 2015) presented four distinct proxemic shapes, namely, concentric circle, egg shape, concentric ellipse, and asymmetric shape. The first, the concentric circle with the human at the center, corresponds to the classic representation proposed by Hall (1966). This symmetric circular formulation does not consider factors such as age, culture, the relationship between interacting agents (human-human or human-robot), and the situational context. The egg shape model introduces asymmetry between the front and back of the human, allocating greater space in the forward direction (Hayduk, 1981). In contrast, the concentric ellipses, proposed by Helbing and Molnár (1995), places the human at the center of an elliptical region (equidistant from the front and back), with a secondary radius defining the lateral extent; this formulation is grounded in pedestrian dynamics and crowd movement studies. The fourth representation, the asymmetric shape, describes a non-symmetrical proxemic zone in which less space is required on the human’s dominant side (usually, the right side) than on the non-dominant side (Gérin-Lajoie et al., 2008). In this model, interpersonal distance is reduced on the pedestrian’s dominant side and remains constant regardless of walking speed (Gérin-Lajoie et al., 2008). Despite their differences, these four shapes primarily represent proxemic zones and provide limited guidance on how proxemic regions might change dynamically in response to factors such as human motion, approach direction, or evolving environmental conditions.

Some studies have reported that in real-world environments individuals often require more space in front of them than in other directions (Hayduk, 1981). This need for a larger frontal distance is attributed to the wide range of actions and social cues that occur in front-facing interactions, such as standing and walking, and engaging in socially mediated behaviors, such as maintaining eye contact. Eye contact can intensify interpersonal interactions and may prevent them from becoming overly intimate; therefore, when an approach occurs from behind, the required personal distance may be reduced (Bailenson et al., 2001). In contrast (Newman Ii and Pollack, 1973), reported opposite findings, showing that individuals sometimes require more space behind them than in other directions. In their study, participants preferred greater distance when approached from the rear compared to frontal or lateral approaches. This preference may be explained by reduced sensory coverage at the back of the body, especially compared to the strong visual sensing available in the front direction.

Our review of the HRI literature related to proxemics (39 papers) indicated that most HRI proxemics models define personal space primarily in scenarios in which a robot approaches a stationary human (20 of the 39 reviewed papers). However, when humans are in motion, additional factors, such as the direction of movement and velocity, become important. Helbing and Molnár (1995) addressed this consideration by modelling the motion of pedestrians through what they call “social forces” that collectively produce an elliptical proxemic shape; these social forces comprise *desired velocity*, which reflects a pedestrian’s preferred speed and direction; *repulsive forces*, which maintain safe distances from other pedestrians and obstacles; and *attractive forces*, which draw pedestrians toward goals or destinations.

A subset of the reviewed studies (7 of 39 papers) explicitly reported asymmetric personal space representations (Daza et al., 2021; Clavero et al., 2022; Neggers et al., 2022a; Kang et al., 2024; Chen and Liu, 2025; Nahum et al., 2025; Spurný et al., 2025). Clavero et al. (2020) proposed a theoretical framework in which proxemics was modeled as an adaptive shape influenced by human activity, location, and culture. This formulation introduced an adaptive cooperation zone that supports natural human-robot collaboration during navigation and interaction tasks; the proxemics zone is represented as an asymmetric Gaussian distribution parametrized by four directional variables: front, back, and lateral sides. Empirical evidence further supports asymmetry in proxemic behavior. Gérin-Lajoie et al. (2008), for example, observed that participants maintained shorter distances on their dominant side when bypassing obstacles. Similar patterns have been observed in corridor navigation, where pedestrians tend to pass others on a preferred side (e.g., the right side), reinforcing the role of lateral asymmetries in spatial behavior. Finally, extending the proxemic zone beyond planar representations, Ribeiro and Macharet (2025) proposed a three-dimensional proxemics model that adds a vertical dimension to the 2D asymmetrical model introduced by Kirby (2010).

Table 1 summarizes the proxemic shape models identified in the reviewed studies, illustrating a shift from static concentric zones to more dynamic, adaptive, context-aware configurations. The reviewed literature reflects a clear theoretical progression from simplified, concentric representations of proxemics (Hall, 1966) toward increasingly context-sensitive and interaction-dependent models (e.g., Clavero et al., 2020). This evolution does not indicate theoretical fragmentation but rather growing consensus that interpersonal comfort cannot be adequately captured by a single, universal geometric shape. Instead, proxemic representations vary as a function of interaction context, task demands, cultural norms, and environmental constraints.

**TABLE 1:** Summary of proxemics shape models in HRI.

Proxemic shape	Geometric characteristics	Key assumptions	Influencing variables considered	Dynamic properties	Limitations
Concentric circles (Hall, 1966)	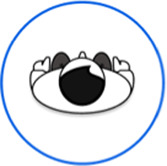	Personal space is uniform in all directions	Culture, age, social relationship, context	Static	Ignores directionality, motion, and environmental dynamics
Egg shape (Hayduk, 1981)	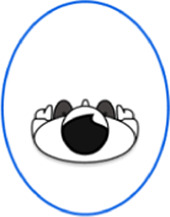	Humans require more space in front	Approach direction, posture, gaze	Mostly static	No lateral asymmetry or velocity modeling
Concentric ellipse (Helbing and Molnár, 1995)	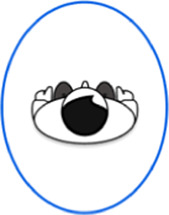	Proxemics emerges from the pedestrian’s dynamics	Human motion, desired velocity, repulsive forces	Dynamic (motion-based)	Limited social interpretation; symmetric laterally
Asymmetric shape (Gérin-Lajoie et al., 2008)	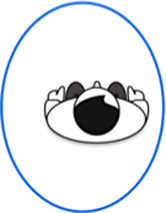	Personal space reflects bodily asymmetry	Dominant hand, passing behavior	Mostly static	Limited treatment of speed, task, and environment
Dynamic proxemic zone (Clavero et al., 2020)	Shape and size vary during interaction	Proxemics adapts to behavior and context	Human behavior, interaction context	Dynamic	Limited modeling of velocity and scene change
3D Proxemics model (Ribeiro and Macharet, 2025)	Three-dimensional asymmetric volume	Personal space extends beyond the 2D plane	Vertical dimension, body height	Dynamic (3D)	Early-stage concept; limited navigation validation

### 2.2 HRI taxonomies and classification

The expanding body of research in HRI has highlighted the need for systematic classification of the variables that shape interaction outcomes. Several studies have thus proposed taxonomies that organize the HRI domain from a complementary perspective. A widely cited framework was introduced by Onnasch and Roesler (2021) to support future HRI research by providing a structured basis for systematically varying key interaction variables. This taxonomy addresses levels of automation, human-automation interaction, and trust in robotic systems, thereby offering a general foundation for classifying HRI characteristics (Onnasch and Roesler, 2021).

Building on application-specific needs, Rodrigues et al. (2023) proposed a multi-dimensional HRI taxonomy tailored to construction tasks. Their framework comprises 16 categories grouped into three clusters: Team (encompassing human and robot roles), Task (addressing task type, planning, and execution), and Environment. This taxonomy is grounded in the assumption that collaborative robotics involves humans and robots working together as a team to interact with their surrounding environment to achieve shared goals.

Focusing specifically on navigation in social contexts, Singamaneni et al. (2024) introduced a taxonomy structured around four classifications of socially aware navigation: robot type, planning and decision-making, situation awareness and assessment, and evaluation methods and tools.

Collectively, these prior classifications emphasize proxemic concepts and define robot variables or specific interaction behaviors, but they do not provide a systematic, integrated taxonomy that jointly accounts for robot traits, human traits, environment, and contextual factors shaping proxemics. These gaps are addressed by our proposed framework.

Kim S. et al. (2024) presented a taxonomy focused on levels and aspects of robot autonomy, providing a structured framework for describing how autonomy is configured and manifested in interaction. Their taxonomy identifies six forms of autonomy: operational, intentional, shared, non-deterministic, cognitive, and physical.

In Section 4, we build on these frameworks to establish a foundational taxonomy of proxemics in HRI, explicitly addressing aspects that remain underrepresented in existing classification schemes. Kim S. et al. (2024) was not included in the taxonomy synthesis because our analysis focuses on classification schemes that organize HRI according to interaction-structural variables directly operationalized in our analytical framework (e.g., role configurations, task structure, interaction context, and planning dimensions).

## 3 Methodology

The study is structured into three phases. First, we identify knowledge gaps in the existing literature by reviewing recent research on proxemics and outlining the key dimensions of proxemics that shape socially aware robot navigation. Next, we derive a structured proxemics taxonomy, utilizing a top-down approach. The overarching hierarchical organization is designed by key dimensions derived from established HRI taxonomies. After manually coding the selected articles across the predefined key dimensions (human, robot, environmental, and contextual factors), we use GenAI to assist in clustering related variables and exploring higher-order conceptual groupings. This taxonomy consists of dimensions (structured aspects that vary across clusters) and variables (elements that can be observed or quantified), influencing the shape, structure, and size of proxemics spaces. Finally, we adopt a bottom-up approach by analyzing and extracting all relevant variables from 39 studies and experiments and integrating the findings into the proposed taxonomy. The outcome is a Taxonomy of Proxemics for Socially Aware Robot Navigation, organized into four clusters: Human, Robot, Environment, and Context as influencing parameters, and the proxemic output variable, an additional Spatial metrics taxon. Each cluster is further divided into categories and variables. This structured taxonomy is aimed to guide future research and practical implementation.

### 3.1 Evaluation and analysis methodology

Our review of studies on robot navigation among people spanned articles appearing in the relevant literature from January 2020 to December 2025, with a focus on HRI research addressing proxemics. Moving beyond a study-by-study evaluation of relevant variables, we implemented a structured variable-classification approach that targeted HRI studies reporting experimental data involving ground robots (mobile wheeled and legged) and humanoid robots across a range of environments, including physical, simulated, and virtual settings (Singamaneni et al., 2024).

Our structured variable-classification methodology identifies research gaps through systematic coding and frequency analysis across studies. Each article was coded along predefined dimensions (human, robot, environmental context, task characteristics, and methodological setting) and aggregated to quantify the prevalence of each variable category across the 2020–2025 corpus.

Understudied areas were operationally defined as domains that appeared in fewer than 5% of studies or were rarely examined in combination with other variables (e.g., multifactorial interactions in real-world environments). Influential dimensions were identified based on their recurrent presence across experimental designs and their consistent association with proxemic variation. Comparing proxemic measurements across variable categories enabled us to determine which human, robot, and contextual factors systematically shaped proxemic zone configurations. This structured cross-tabulation provided a transparent basis for identifying both concentrations and gaps in the literature.

**FIGURE 1:**
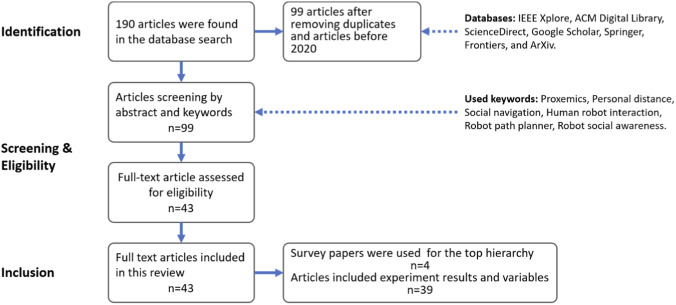
Diagram of the article search and screening process for inclusion in the proxemics evaluation.

The following three stages were applied for scoping and conducting the literature review: Identification, Screening & Eligibility, and Inclusion (Figure 1).

**FIGURE 2:**
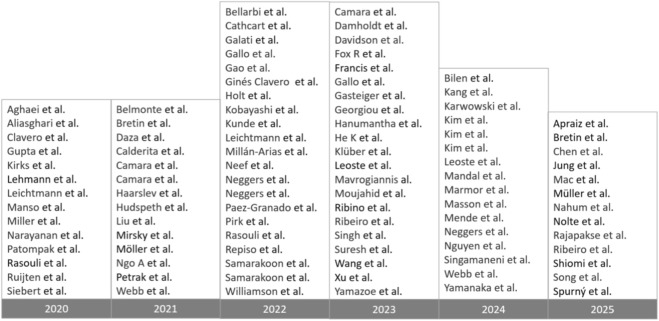
Distribution by years (January 2020 to December 2025) of social robot navigation papers focusing on proxemics (complete reference details can be found in the Supplementary Materials).

- *Identification:* For the literature search, the following query keywords were used: Proxemics, Personal distance, Social navigation, Human-robot interaction, Robot path planner, and Robot social awareness. We sourced papers and articles on human-robot proxemics through the following databases: IEEE Xplore, Springer, ACM Digital Library, ScienceDirect, Google Scholar, Frontiers, and ArXiv. The literature library compiled as part of this review contains scientific publications, magazine articles, conference publications, and theses. The process yielded more than 190 articles in the initial search. Thereafter, we excluded duplicated articles and articles published before January 2020.
- *Screening and eligibility:* We then screened all articles by abstract and keywords. The screening criteria included: proxemics, personal distance, social navigation, HRI, robot path planner, and robot social awareness. This yielded 99 articles (Figure 2). From these, all theoretical and opinion papers were removed, yielding 43 articles consisting of empirical studies or literature surveys.
- *Inclusion:* The 43 papers were organized into two groups (Figure 1): four survey articles (Section 2) were used to derive key variables for the proxemics top hierarchy, and 39 experimental articles (Section 5) were used to extract proxemics variables.

Our analysis mapped the parameters and variables that influence HRI proxemics, emphasizing their dynamic and context-dependent nature. Two guidelines steered our analysis. First, we examined the experimental methodologies used to define and evaluate proxemic shapes. Second, we identified the key variables shaping proxemic behavior and characterized them through both quantitative and qualitative analyses. Altogether, this process enabled us to identify commonalities and gaps in existing proxemic definitions and experimental practices.

### 3.2 Variables influencing the shape of personal space

**FIGURE 3:**
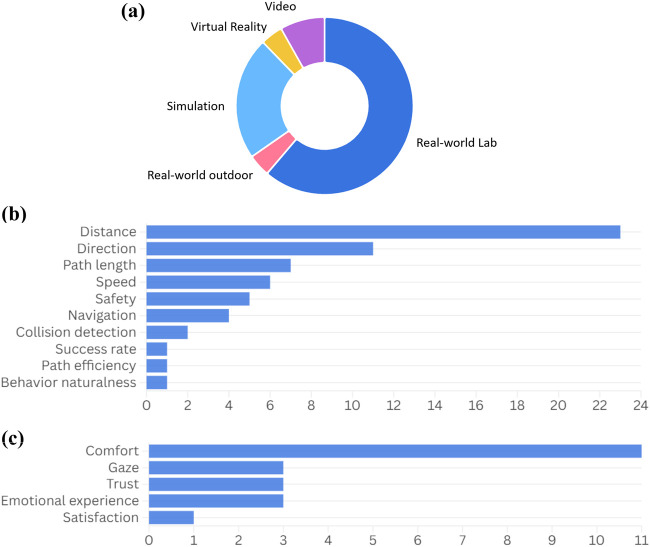
Distribution in the reviewed studies of **(a)** environmental conditions; **(b)** performance metrics; and **(c)** social-related metrics.

The shape of personal space is influenced by multiple factors, including robot speed and movement direction, individual characteristics, social cues, and task content. Task content refers to the specific nature and requirements of the activity performed by a human or a robot, and represents a critical factor, as proxemics behavior varies with the actions being carried out. User studies are essential for understanding how these factors affect proxemics, safety, and task performance. Existing studies differ substantially in their experimental settings, including variations in human and robot conditions, environments, and evaluation metrics. The following summary (Figure 3) highlights key differences across study designs, environments and metrics they employ.

- *Human position and robot approach*: In 19 studies, humans were examined in static positions, while in 11, they were captured while walking, and in 9 both positions were examined. In most studies (23), only a single proxemics-related variable was tested at a time (e.g., human posture or approach direction). Six studies investigated two parameters simultaneously (e.g., the participant standing or sitting while the robot approaches from multiple directions), and only five papers analyzed more than two parameters, such as: human position, robot velocity, and approach direction (Ruijten and Cuijpers, 2020; Haarslev et al., 2021; Neggers et al., 2022a; Repiso et al., 2022; Yamazoe et al., 2023).
- *Environment*: Most of the studies (30) were conducted in controlled laboratory settings which, while methodologically robust, lacked ecological validity. Real-world outdoor experiments were rare, appearing only in two studies (Repiso et al., 2022; Chen and Liu, 2025). Additionally, two studies were conducted in virtual environments (Klüber and Onnasch, 2023; Jung et al., 2025), eleven were performed in simulated environments, and four evaluated robot behavior using video-based scenarios from an observer’s perspective (Aliasghari et al., 2020; Petrak et al., 2021; Gallo et al., 2022; He et al., 2023). These distributions are summarized in Figure 3a.
- *Metrics*: Most studies relied on efficiency and safety metrics, including distance (23 studies), approach direction (11), speed (6), path length (7), safety indicators (5), navigation performance (4), success rate (1), path efficiency (1), collision detection (2) (Figure 3b). In contrast, social-related metrics, such as gaze (3), comfort (11), trust (3), emotional experience (3), satisfaction (1), and perceived behavior naturalness (1), were used less frequently and often served as indirect indicators of safety or overall user perception (Figure 3c).

In summary, proxemics research in socially aware robot navigation is dominated by indoor laboratory experiments, with relatively few studies conducted in real-world outdoor environments. Most studies focus on a single variable rather than on multiple variables, highlighting the need for more comprehensive, multifactorial studies in ecologically valid settings.

## 4 Developing a taxonomy of proxemics in HRI

In this section, we bridge the gap between theoretical concepts derived from HRI models (Section 4.1) and variables reported in proxemics-related HRI studies, including both physical and simulated experiments (Figure 3). Proxemics refers to the spatial relations that regulate interaction, encompassing interpersonal distance, relative orientation, and movement within a shared space. Accordingly, factors such as robot morphology, human characteristics, environmental settings, and context are treated as influencing variables that shape proxemic behavior rather than proxemic constructs themselves.

The development of the proxemics taxonomy follows two complementary and parallel pathways designed to integrate conceptual, computational, and empirical perspectives.

The top-down pathway combines theory-driven analysis with AI-assisted knowledge structuring. First, we systematically derive proexmics-relevant constructs from existing HRI taxonomies that classify interactions according to structural and interactional variables, such as roles, task structure, interaction context, and planning characteristics, based on the variables identified in Section 2.2 (Onnasch and Roesler, 2021; Rodrigues et al., 2023; Singamaneni et al., 2024). Rather than adopting these taxonomies directly, we reinterpret and map their constructs into proxemics phenomena, extracting those dimensions that meaningfully regulate spatial interaction. Each taxonomy is analyzed independently in Section 4.1. In parallel, an AI-assisted exploration is employed as a generative knowledge-structuring tool (Section 4.2), producing an alternative data-driven taxonomy that enables comparison with theory-driven constructs and reveals potential high-level proxemics constructs not explicitly represented in HRI frameworks to identify absent from current HRI taxonomies.

The bottom-up pathway (Section 4.3) complements this process by deriving variables directly from reported studies and experiments.

**FIGURE 4:**
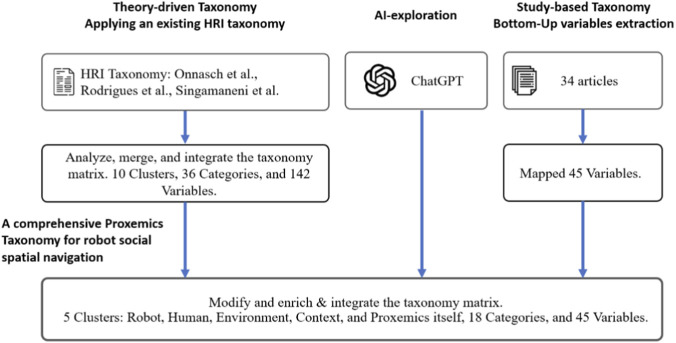
Methodological flow for generating the proxemics taxonomy.

This empirical synthesis grounds the resulting taxonomy (Figure 4) in observed proxemics behaviours and allows convergence between theory-derived constructs, AI-generated structure and, evidence emerging from the literature.

### 4.1 Applying a theoretical HRI taxonomy: top-down approach

**FIGURE 5:**
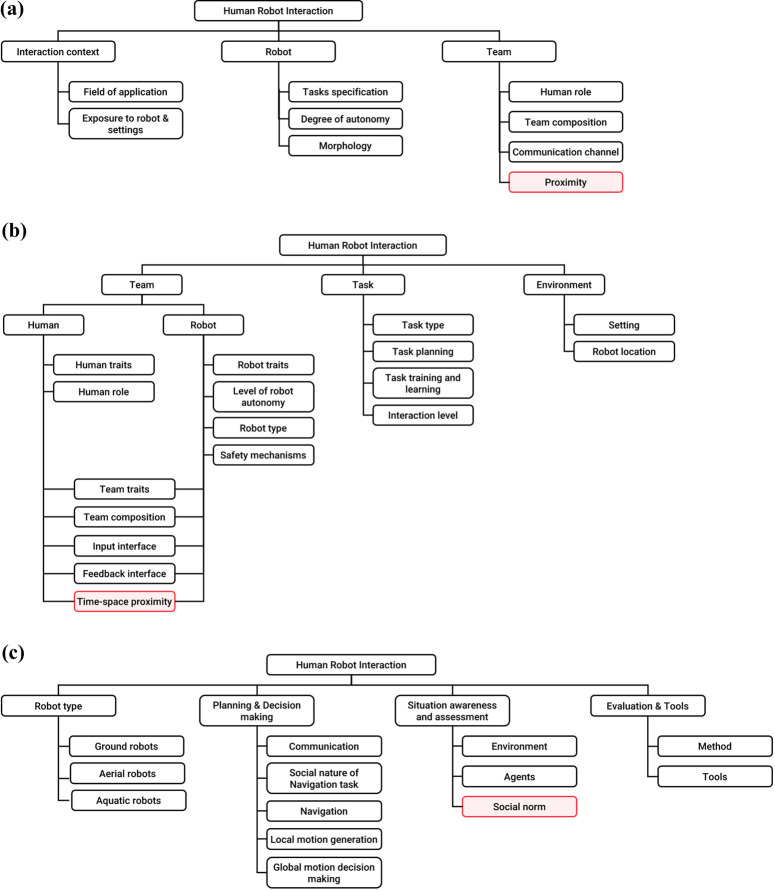
Overviews of HRI taxonomies with highlighted Proxemics-related elements (colored red), according to **(a)** Onnasch and Roesler (2021); **(b)** Rodrigues et al. (2023); and **(c)** Singamaneni et al. (2024).

Three theoretical taxonomy articles provided a systematic classification of HRI studies, with each article approaching the domain from a different perspective. These works address broader dimensions of the HRI taxonomy, with proxemic aspects included only partially or indirectly. Each taxonomy (Figure 5) explicitly identifies where proxemic-related variables appear and distinguishes them from non-proxemic factors. While some variables, such as robot morphology, safety, or communication, are not proxemics *per se*, they are included in the taxonomy because they systematically shape, constrain, or modulate proxemic behavior. For example, Neggers et al. (2022a) and He et al. (2023) illustrate how variables such as robot morphology influence proxemic behavior through their effects on user responses, including perceived comfort and distance regulation.

Onnasch and Roesler (2021) proposed a multi-dimensional framework organized into three clusters: interaction context, robot, and team (Figure 5a). The interaction context cluster defines the domain of interaction and includes two categories: the field of application, related to different robot types (service, industrial, etc.), and exposure to the robot, including whether interaction occurs physically or virtually and the characteristics of the study setting. The robot cluster classifies robot-based interaction-related variables, focusing on the robot’s work context and design. This cluster includes robot task specification, degree of autonomy, and robot morphology. The team cluster captures variables relevant to HRI scenarios involving multiple agents, addressing the interaction structure, the team composition, and collaboration. These dimensions are defined by four variables: human role, team composition, communication channel, and proximity.

Rodrigues et al. (2023) introduced a multi-dimensional HRI taxonomy specifically tailored to construction tasks (Figure 5b). This taxonomy is structured around three dimensions, team, tasks, and environment, where: (a) team encompasses both human and robot participants, (b) tasks addresses task type, planning, and execution mechanisms, and (c) environment accounts for contextual variables influencing collaboration. The taxonomy is grounded in the premise that collaborative robotics involves humans and robots working jointly as a team within a shared environment to accomplish a given task. In total, 17 categories are organized across these three clusters.

Singamaneni et al. (2024) proposed a taxonomy focused on socially aware navigation, organized around four perspectives: robot type, planning and decision-making, situation awareness and assessment, and evaluation and tools (Figure 5c). These perspectives are represented through four clusters comprising a total of 13 categories, offering a structured view of the technical, cognitive, and evaluative aspects of socially aware robotic navigation.

Subsequently, we synthesize these frameworks and derive from them an HRI proxemics taxonomy that provides a structured overview of existing research. To achieve this, we first extracted the hierarchical levels and variables from each taxonomy and consolidated them into a unified dataset. This initial aggregation yielded 142 variables, mapped across 36 categories, and organized into 10 clusters.

We then identified and resolved similarities, overlaps, and duplications among taxonomy levels and variables. Through a systematic process of terminology harmonization and variable alignment, the taxonomy was refined to 126 variables mapped to 33 categories and eight clusters.

**FIGURE 6:**
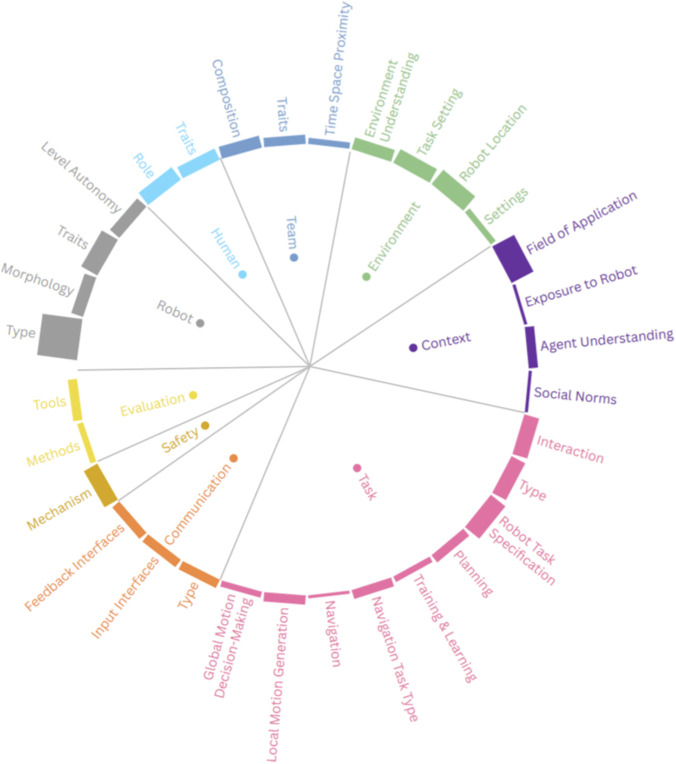
Visualization of the integrated and verified HRI taxonomy structure from (Onnasch and Roesler, 2021; Rodrigues et al., 2023; Singamaneni et al., 2024). The height of the blocks represents the number of variables contained in each category. Each color represents a different cluster and is used consistently throughout the article. *Colors correspond to the colors in Table 2 and Figure 8 (robot - gray, human - shades of blue, environment - green, context - purple, and Spatial metrics - red).

The final verified taxonomy, presented in Figure 6, captures both the distinct perspectives of the original frameworks and their underlying conceptual foundations, while highlighting common clustering patterns across HRI taxonomy mappings. Certain components of HRI mapping, such as evaluation, safety, and communication, do not directly determine interpersonal distance. Instead, they are included because they constrain or mediate proxemic behavior by shaping human perception or defining operational limits for robot motion. In contrast, planning, navigation, and decision-making have a more direct relationship to proxemics, as these processes govern the robot’s spatial positioning and movement trajectories relative to human counterparts.

### 4.2 Exploratory AI tool: a complementary top-down approach

To ensure we provide an extensive mapping of our proposed proxemics taxonomy, we explored the use of a generative AI tool, specifically ChatGPT 4.0 to complement the HRI taxonomies derived from above.

Generative AI was used strictly as an analytical support tool during the synthesis phase of our structured literature review. After manually coding the selected articles across predefined clusters (human, robot, environmental, and contextual factors), we used GenAI to assist in clustering related variables and exploring potential higher-order conceptual groupings. The tool did not generate independent theoretical constructs and was not used as a source of empirical claims or textual content.

All categories, labels, and relationships were derived from the reviewed studies and were validated through repeated cross-checking with the primary literature. Conceptual groupings suggested during the AI-assisted clustering process were retained only when supported by multiple cited sources. No AI-generated statements were included without verification, and all citations were independently identified and confirmed by the authors.

**FIGURE 7:**
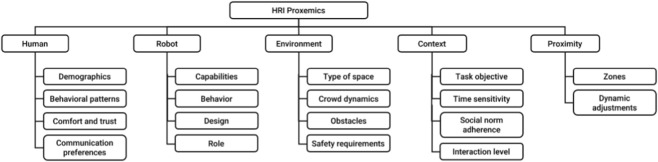
Generative AI (ChatGPT) exploratory outcomes for the query to create an HRI Proxemics taxonomy.

The system was first prompted with the query: “Can you provide a structured HRI proxemics taxonomy, organized hierarchically based on Human, Robot, Environment, and Context?” Then, to narrow the first query’s results to focus on proxemics, it was followed by a refined prompt: “Can you provide it for HRI proxemics?” The resulting output proposed five clusters, each populated with detailed categories and associated variables (Figure 7).

A comparison between the AI-suggested taxonomy and the theoretically derived mapping revealed, as expected, a high degree of similarity in the clustering of human, robot, environment, and context-related dimensions. The primary difference was the emergence in the AI-generated taxonomy of an additional cluster explicitly dedicated to proxemics. This differentiation is conceptually meaningful, as existing theoretical HRI taxonomies do not explicitly foreground proxemics, even though the spatial configuration of interactions can be understood as possessing distinct characteristics. Together, these two approaches established a top-down perspective that outlined the anticipated structural framework of the taxonomy, which subsequentially served as the foundation for bottom-up validation.

### 4.3 Bottom-up article-survey-driven approach

To complement the above analysis, a bottom-up study-driven analysis was applied to examine whether the above clusters emerge organically from observed interactions and user responses, thereby enabling the validation, refinement, and/or extension of the proposed taxonomy based on real-world evidence. A manually curated review of all 39 articles that focused on proxemics and contained experimental variables yielded 45 variables. These variables were grouped into categories and clusters, based on the theoretical (Top-Down) structure. This process revealed variables that were not covered in the existing theoretical mapping, such as human skill and experience, which led to the definition of an additional category.

When this classification was applied to HRI proxemics from a top-down perspective, it became evident that the taxonomies proposed in the HRI literature (Onnasch and Roesler, 2021; Rodrigues et al., 2023; Singamaneni et al., 2024) do not fully capture the diversity of variables examined in the proxemics-related studies reviewed in Section 2.2. Several important dimensions are absent from the existing taxonomies, including human personal data and physical characteristics, environmental constraints, temporal and spatial dynamics, and behavioral variables. In addition, further variables emerge from the literature that are not explicitly represented in current classifications, such as task planning, task training, and learning processes (Table 2).

**TABLE 2:** Comparison between the combined theoretical HRI taxonomy, the GenAI exploratory taxonomy, and the suggested proxemics taxonomy derived from the bottom-up data-driven survey.

HRI taxonomies		GenAI exploratory taxonomy		Proxemics taxonomy	
Cluster	Category	Cluster	Category	Cluster	Category
Robot	Type	Robot	Design	Robot	Design
	Morphology				Size
	Traits		Behavior		Behavior
	Level of autonomy		Capabilities		Autonomy
			Role		Task
Human	Role	Human	Demographics	Human	Personal data
	Traits		Behavioral patterns		Activity
					Physical
					Context
Team	Composition				
	Traits				
	Time-space proximity			Context	Collaboration
Environment	Environment understanding	Environment	Type of space	Environment	Indoor
					Outdoor
	Task setting**				
	Robot location				
	Settings				
Context	Field of application				
	Exposure to robot			Context	Robot
	Agent understanding				
	Social norms	Context	Social norm adherence	Spatial metrics	Shape
			Time sensitivity		Morphing
				
Task	Interaction	Context	Interaction level		
	Type			Context	Robot
	Robot task specification	Context	Task objective	Context	Robot
	Planning				
	Training & Learning				
	Navigation			Context	Robot
	Local motion generation				
	Global motion decision-making				
Communication	Communication	Human	Communication preferences		
	Input interfaces				
	Feedback interfaces				
	Communication channel				
	Physical proximity			Robot	Task
Safety	Mechanism	Environment	Safety requirements		
Evaluation	Methods			Environment	Simulation
					Real world
					Virtual Reality
					Video
	Tools				
		Proximity	Zones	Spatial metrics	Shape
			Dynamic adjustments		Measurement
					Morphing

**FIGURE 8:**
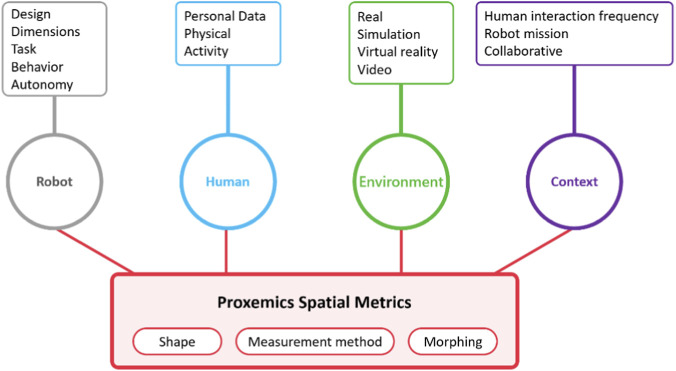
Overview of the proxemics taxonomy (each color represents a different cluster: robot - gray, human - shades of blue, environment - green, context - purple, and spatial metrics - red).

*Colors correspond to the colors in Figures 6, 8 (robot - gray, human - shades of blue, environment - green, context - purple, and Spatial metrics - red). ** Task setting (per Rodrigues et al.) was retained as a subcategory within the Environment cluster because the variables it contained (e.g., on-site, off-site, laboratory) describe the physical or experimental setting in which the interaction occurred, rather than the task itself.

## 5 Proposed integrated taxonomy of proxemics

This section describes how the theoretical top-down approach was integrated with findings from the empirical studies that used a bottom-up approach to form a unified taxonomy. First, we established the top-level taxonomy clusters by identifying three core clusters: *Human*, *Robot*, and *Environment*, which appear in varying forms across the theoretical literature (Figure 8) and the AI-generated taxonomy. Next, we consolidated context-related variables that were derived from the bottom-up approach scattered across different studies and organized them into a dedicated *Context* cluster. This cluster is essential for understanding the purpose of the interaction and how that purpose influences the personal distance. These four clusters define the influencing parameters that shape proxemic behavior. Finally, we introduced a distinct *Spatial metrics* output variable that encompasses all properties related to proxemic characteristics.

Notably, most studies (22) examined only a single variable, such as robot height (1), human position (15), or robot emotions (1), while a smaller subset analyzed two variables (14), including approach directions (8), velocity (4), or the presence of individuals vs. groups (5). These variables collectively informed the definition of the proposed set of taxa. The resulting taxonomy structure, presented below in Sections 5.1-5.5, reflects the integration of our theoretical and empirical findings.

The proposed taxonomy mapping is intended to consolidate and restructure existing variables explicitly through a proxemic perspective. Rather than introducing new constructs, it systematically integrates variables that have previously appeared in dispersed or implicitly proxemic form across the literature. While broad labels (such as personal, physical, and context) may appear abstract, this level of generality is intentional, enabling the taxonomy to synthesize findings across heterogeneous HRI studies. Each category encompasses more specific variables identified in the review (e.g., robot dimensions and morphology under physical variables; environmental objects and spatial layout under context), thereby providing a structured framework for interpreting proxemic influences.

### 5.1 Taxonomy variables for the robot cluster

**FIGURE 9:**
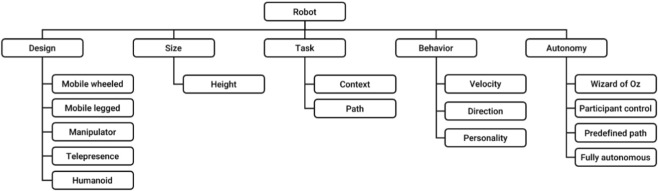
Taxonomy for robot characteristics, tasks, behavior, and control.

Figure 9 and Table 3 present the various variables related to the robot that shape the proxemics behavior. As noted, our analyses focused exclusively on articles involving mobile ground robots (robots that operated and move on the ground). Among the reviewed studies, mobile robots were used in 21 cases and humanoid robots in 13. Direction emerged as measurable variable in most studies, appearing in 14 of them.

**TABLE 3:** Attribution of the papers reviewed to the robot taxa for design, size, task, behavior, and autonomy.

Term	Definition	Articles
Design	This taxon defines the robot type and embodiment characteristics	
Mobile wheels	Any robot that moves on wheels and looks like a machine	Kirks et al., 2020; Siebert et al., 2020; Daza et al., 2021; Haarslev et al., 2021; Bellarbi et al., 2022; Cathcart et al., 2022; Neef et al., 2022; Neggers et al., 2022a, 2022b; Gallo et al., 2022; Samarakoon et al., 2022a; Gallo et al., 2023; Klüber and Onnasch, 2023; Yamazoe et al., 2023; Kang et al., 2024; Kim Y. S. et al., 2024; Nguyen and Ngo, 2024; Chen and Liu, 2025; Jung et al., 2025; Nahum et al., 2025; Spurný et al., 2025
Mobile legs	Any robot that moves on legs	Xu et al., 2023; Spurný et al., 2025
Manipulator	A mobile robot designed to perform physical manipulation tasks with its arm	Miller et al., 2020; Clavero et al., 2022; Hanumantha, 2023; He et al., 2023
Telepresence	Any robot that enables users to maintain a virtual presence in a remote location. The user can control the robot’s movements and other behaviors through a computer	Neef et al., 2022; Leoste et al., 2023; Singh et al., 2023; Marmor et al., 2024; Nahum et al., 2025
Humanoid	A robot shaped like a human (body and head, sometimes even hands)	Neggers et al., 2018; Aliasghari et al., 2020; Lehmann et al., 2020; Patompak et al., 2020; Ruijten and Cuijpers, 2020; Petrak et al., 2021; Liu et al., 2021; Neggers et al., 2022a, 2022b, 2024; Repiso et al., 2022; Moujahid et al., 2023; Müller and Richert, 2025
Size	This taxon defines the robot’s physical measurements	
Height	The measurement from the floor to the highest point of the robot	Miller et al. (2020)
Task	This taxon contains goals or functional objectives that the robot must perform during interaction	
Context	The scene conditions and the situation in which the interaction occurs	Miller et al., 2020; Daza et al., 2021; Liu et al., 2021; Gallo et al., 2022; Samarakoon et al., 2022a; Leoste et al., 2023; Moujahid et al., 2023; Singh et al., 2023; Marmor et al., 2024; Jung et al., 2025; Müller and Richert, 2025; Spurný et al., 2025
Path	The trajectory from the origin to the target point	He et al. (2023)
Behavior	This taxon defines the specific actions and movement patterns a robot employs during an interaction	
Velocity	The speed of movement that is related to both task priority and scene limitations	Neggers et al., 2022b, 2024; Klüber and Onnasch, 2023; Yamazoe et al., 2023; Chen and Liu, 2025; Jung et al., 2025)
Directions	The side/angle at which a robot approaches the person for interaction	(Ruijten and Cuijpers, 2020; Siebert et al., 2020; Liu et al., 2021; Neef et al., 2022; Neggers et al., 2022a, 2022b, 2024; He et al., 2023; Xu et al., 2023; Yamazoe et al., 2023; Nguyen and Ngo, 2024; Chen and Liu, 2025; Jung et al., 2025; Nahum et al., 2025
Personality	A reflection of human emotional patterns, such as happy, angryetc.	Moujahid et al. (2023)
Autonomy	This taxon defines the robot’s capacity to independently perceive its environment and execute spatial movements	
Wizard of Oz	When an operator controls the robot behind the scenes, with the participant being unaware of the operator control	Miller et al., 2020; Liu et al., 2021; Neef et al., 2022; Gallo et al., 2023; Marmor et al., 2024
Participant control	When the participant moves the robot with a remote control during the experiment	Leoste et al., 2023; Kim Y. S. et al., 2024
Predefined path	When a robot moves between two predefined points, the starting point and the destination	Ruijten and Cuijpers, 2020; Haarslev et al., 2021; Cathcart et al., 2022; Klüber and Onnasch, 2023; Yamazoe et al., 2023; Kang et al., 2024; Neggers et al., 2024; Spurný et al., 2025
Fully autonomous	When a robot that can sense and move independently among humans in the same environment	Lehmann et al., 2020; Siebert et al., 2020; Bellarbi et al., 2022; Neef et al., 2022; Neggers et al., 2022a, 2022b; Repiso et al., 2022; Samarakoon et al., 2022a; Singh et al., 2023; Xu et al., 2023

### 5.2 Taxonomy variables for the human cluster

**FIGURE 10:**
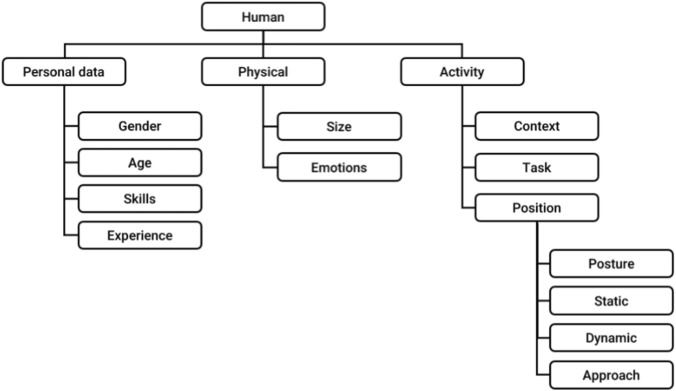
Taxonomy for human characteristics and behavior.

Figure 10 and Table 4 present the variables related to personal, physical, and activity-related aspects of the human. A static human pose was used in 24 studies, while 14 studies examined dynamic scenarios. Only eight studies collected personal characteristics as measured variables, and none considered culture as a variable in defining proxemics, despite its prominence in earlier literature.

**TABLE 4:** Attribution of the papers reviewed to human taxa for personal data, physical properties, and activity.

Term	Definition	Articles
Personal data	This taxon categorizes individual sociodemographic traits and user experience	
Gender	Gender affects human behavior	Klüber and Onnasch, 2023; Leoste et al., 2023; Xu et al., 2023
Age	Age affects human physical dimensions and behaviors	Kang et al. (2024)
Skill	If the human has a specific experience that differentiates him/her from others	Hanumantha, 2023; Klüber and Onnasch, 2023; Leoste et al., 2023
Experience	If the participant has previous experience with robots	Miller et al. (2020)
Physical	This taxon contains the physical measurements and human traits	
Size	The human height that, in most cases, is derived from age	Xu et al., 2023; Kang et al., 2024
Emotions	Human mood affects social behavior	Petrak et al. (2021)
Activity	This taxon captures the human’s current task or behavioral state	
Context	When an interaction is based on a real-world scenario, such as the delivery of an item	Ruijten and Cuijpers, 2020; Haarslev et al., 2021; Repiso et al., 2022; Gallo et al., 2023; Leoste et al., 2023; Moujahid et al., 2023; Marmor et al., 2024; Jung et al., 2025
Task	Interaction occurs when the human has a specific activity or mission to accomplish	Xu et al., 2023; Kim Y. S. et al., 2024
Position	This taxon includes the physical state of the human, it’s posture and body orientation	
Posture	Body poses during the interaction, such as standing or sitting	Samarakoon et al., 2022a; Klüber and Onnasch, 2023; Nahum et al., 2025; Spurný et al., 2025
Static	When the human is positioned at the same stationary point during the experiment	Aliasghari et al., 2020; Kirks et al., 2020; Miller et al., 2020; Patompak et al., 2020; Ruijten and Cuijpers, 2020; Daza et al., 2021; Haarslev et al., 2021; Liu et al., 2021; Petrak et al., 2021; Bellarbi et al., 2022; Clavero et al., 2022; Neef et al., 2022; Neggers et al., 2022a; Repiso et al., 2022; Gallo et al., 2022; Samarakoon et al., 2022a; Gallo et al., 2023; Hanumantha, 2023; Klüber and Onnasch, 2023; Leoste et al., 2023; Singh et al., 2023; Yamazoe et al., 2023; Kang et al., 2024; Kim Y. S. et al., 2024; Marmor et al., 2024; Jung et al., 2025; Nahum et al., 2025; Spurný et al., 2025
Dynamic	When the human moves from the experiment’s starting point by walking or changing position	Lehmann et al., 2020; Siebert et al., 2020; Haarslev et al., 2021; Cathcart et al., 2022; Neggers et al., 2022b, 2024; Repiso et al., 2022; Gallo et al., 2022; Samarakoon et al., 2022a; Hanumantha, 2023; He et al., 2023; Leoste et al., 2023; Moujahid et al., 2023; Singh et al., 2023; Xu et al., 2023; Kim Y. S. et al., 2024; Nguyen and Ngo, 2024; Chen and Liu, 2025; Müller and Richert, 2025; Spurný et al., 2025
Approach	When a human nears a robot in preparation for an interaction	Lehmann et al., 2020; Moujahid et al., 2023; Müller and Richert, 2025; Nahum et al., 2025; Spurný et al., 2025

### 5.3 Taxonomy variables for the environment cluster

**FIGURE 11:**
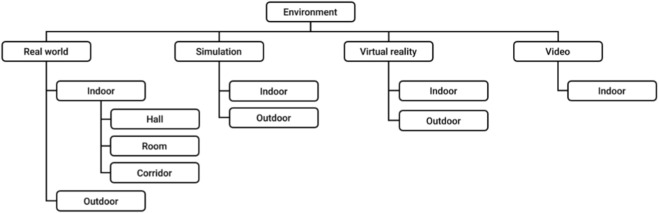
Taxonomy variables for the environment.

Figure 11 and Table 5 present the environment variables related to the study setting and spatial characteristics of the interaction, including scene type and its associated variables. Most studies were conducted in laboratory settings (30), reflecting highly controlled experimental conditions, while only two experiments were performed outdoors.

**TABLE 5:** Attribution of the papers reviewed to robot and human taxa for environment type.

Term	Definition	Articles
Real-world	This taxon contains studies that conducted experiments in a physical setting with participants and robots	
Indoors	• Hall: An open space characterized by a large size • Room: a small space blocked out by walls, which can be a lab or teaching room • Corridor: an indoor lane used to move between places; it is blocked out by walls on its sides, and has an average width of 0.9–1.2 m	Aliasghari et al., 2020; Kirks et al., 2020; Lehmann et al., 2020; Miller et al., 2020; Patompak et al., 2020; Ruijten and Cuijpers, 2020; Siebert et al., 2020; Haarslev et al., 2021; Liu et al., 2021; Bellarbi et al., 2022; Cathcart et al., 2022; Clavero et al., 2022; Neef et al., 2022; Neggers et al., 2022b, 2022a, 2024; Samarakoon et al., 2022a; Gallo et al., 2023; He et al., 2023; Leoste et al., 2023; Moujahid et al., 2023; Singh et al., 2023; Xu et al., 2023; Yamazoe et al., 2023; Kang et al., 2024; Marmor et al., 2024; Chen and Liu, 2025; Müller and Richert, 2025; Nahum et al., 2025; Spurný et al., 2025
Outdoors	• Sidewalk: a public pathway shared with other people • Public space: an open public space	Repiso et al., 2022; Chen and Liu, 2025
Simulation	This taxon groups all studies set in a computer-based simulation environment	
Indoors	Same as real-world indoors	Patompak et al., 2020; Daza et al., 2021; Haarslev et al., 2021; Bellarbi et al., 2022; Clavero et al., 2022; Hanumantha, 2023; Kang et al., 2024; Kim Y. S. et al., 2024; Nguyen and Ngo, 2024; Chen and Liu, 2025
Outdoors	Same as real-world outdoors	Repiso et al. (2022)
Virtual reality	This taxon contains experiments that allow the researcher to isolate the participant from environmental interference with the complete scene	
Indoors	A closed space blocked out between walls and a ceiling	Klüber and Onnasch (2023)
Outdoors	For an open public space scene	Jung et al. (2025)
Video	This taxon contains experiments conducted while a participant watches video clips and subsequently gives feedback	
Indoors	A closed space is blocked out between the walls and the ceiling	Aliasghari et al., 2020; Petrak et al., 2021; Gallo et al., 2022; He et al., 2023)

We treat modality as a methodological cluster that shapes how proxemic phenomena are captured and interpreted, as it influences how proxemic behavior is measured, perceived, and operationalized in empirical studies. Similarly, the indoor-outdoor distinction is included as a macro-contextual variable that recurs throughout the reviewed corpus, reflecting a structural environmental contrast between controlled laboratory settings and dynamic public spaces.

### 5.4 Taxonomy variables for the context cluster

**FIGURE 12:**
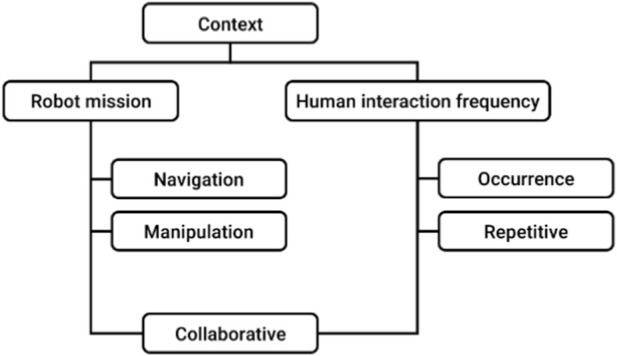
Taxonomy variables for human role and robot role contexts.

Figure 12 and Table 6 present contextual aspects of the reviewed studies. This classification encompasses experiments in which situational variables, tasks, and goals are tied to real-world situations. Single human tasks were identified in 10 studies, while robot navigation was the most frequently reported task (21). Human-robot collaboration was rarely addressed, appearing in only four studies.

**TABLE 6:** Attribution of the papers reviewed to robot and human taxa for context.

Term	Definition	Articles
Robot mission	This taxon contains the robot’s goal or task to achieve	
Navigation	When a robot moves through space from one waypoint to another	Aliasghari et al., 2020; Miller et al., 2020; Ruijten and Cuijpers, 2020; Haarslev et al., 2021; Cathcart et al., 2022; Clavero et al., 2022; Neef et al., 2022; Neggers et al., 2022a, 2022b; Repiso et al., 2022; Samarakoon et al., 2022a; Gallo et al., 2023; Hanumantha, 2023; He et al., 2023; Klüber and Onnasch, 2023; Singh et al., 2023; Yamazoe et al., 2023; Kang et al., 2024; Kim Y. S. et al., 2024; Jung et al., 2025; Spurný et al., 2025
Manipulation	The robot has a mission to perform some activity	Siebert et al., 2020; Bellarbi et al., 2022
Both	This taxon is for the robot and human collaboration task	
Collaboration	A task shared by a human and a robot	Lehmann et al., 2020; Gallo et al., 2022; Moujahid et al., 2023; Marmor et al., 2024
Human interaction frequency	This taxon is for the human interaction task frequency	
Single	Task that occurred once during the interaction	Patompak et al., 2020; Ruijten and Cuijpers, 2020; Haarslev et al., 2021; Bellarbi et al., 2022; Cathcart et al., 2022; Samarakoon et al., 2022a; Gallo et al., 2023; He et al., 2023; Singh et al., 2023; Kim Y. S. et al., 2024
Repetitive	A task that occurs several times during the interaction	Miller et al., 2020; Liu et al., 2021

### 5.5 Spatial metrics taxon of proxemics

**FIGURE 13:**
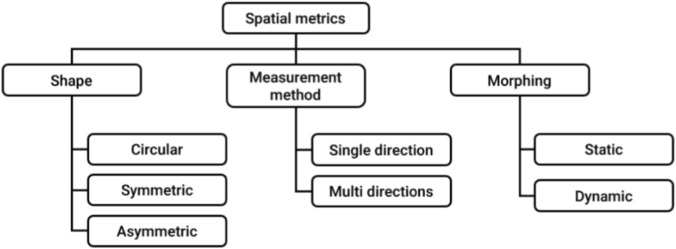
Spatial metrics variables that directly influence proxemics shape, measurement method, and morphing.

All above variables in the four clusters (robot, human, environmental, and contextual) shape the proxemics spatial metrics, this taxon defines the proxemics output variables. Figure 13 and Table 7 summarize experiments that specifically measured proxemic characteristics. This includes the shape of the proxemics zone (7 employed an asymmetric proxemic shape, while nine used Hall’s traditional shape) and the measurement direction (8 studies measured from a single direction 8 and 11 studies measured from multiple directions). The proxemics shape was static in most studies (11), with only two studies including a dynamic shape.

**TABLE 7:** Attribution of the review papers to robot taxa for the proxemics-related spatial metrics.

Term	Definition	Articles
Shape	This taxon defines the geometry of the proxemic space footprint on the ground	
Circular	A circular shape based on Hall’s classification	Aliasghari et al., 2020; Kirks et al., 2020; Haarslev et al., 2021; Samarakoon et al., 2022a; Hanumantha, 2023; Singh et al., 2023; Nguyen and Ngo, 2024; Jung et al., 2025; Müller and Richert, 2025
Symmetric	A symmetric shape when the person is located in the center	Torta et al., 2012; Daza et al., 2021; Chen and Liu, 2025
Asymmetric	An oval shape: the participant is in the middle, and the distances from him/her to the shape boundaries are not equal	Patompak et al., 2020; Clavero et al., 2022; Neggers et al., 2022a; Kang et al., 2024; Chen and Liu, 2025; Nahum et al., 2025; Spurný et al., 2025
Measurement method	This taxon describes the measurement method of variables that define the proxemic shape	
Single direction	A one-directional measurement is taken to define the shape, usually the forward distance	Lehmann et al., 2020; Miller et al., 2020; Samarakoon et al., 2022a; Klüber and Onnasch, 2023; Leoste et al., 2023; Moujahid et al., 2023; Kim Y. S. et al., 2024; Marmor et al., 2024
Multi-directional	More than one direction is measured to define the proxemics shape	Ruijten and Cuijpers, 2020; Siebert et al., 2020; Liu et al., 2021; Cathcart et al., 2022; Neef et al., 2022; Neggers et al., 2022a, 2022b; Xu et al., 2023; Yamazoe et al., 2023; Kang et al., 2024; Nahum et al., 2025
Morphing	This taxon describes the transformation of the proxemic shape	
Static	A predefined fixed shape whose size does not change during the interaction	Torta et al., 2012; Kirks et al., 2020; Patompak et al., 2020; Daza et al., 2021; Haarslev et al., 2021; Bellarbi et al., 2022; Clavero et al., 2022; Neggers et al., 2022a; Hanumantha, 2023; Kang et al., 2024; Nguyen and Ngo, 2024
Dynamic	A shape that changes size during the interaction and in response to contextual and situational factors	Samarakoon et al., 2022a; Spurný et al., 2025

## 6 Discussion

In this study, we propose a proxemics taxonomy for human robot interaction (HRI) derived from the integration of complementary theoretical and empirical perspectives, addressing key gaps in existing frameworks for socially aware robot navigation. The methodology combines top-down and bottom-up analytical approaches to define relevant proxemic constructs and variables.

The top-down pathway draws on a critical examination of established HRI taxonomies alongside an AI-assisted exploratory analysis, which together generate a comprehensive set of candidate theoretical constructs describing spatial interaction. In contrast, the bottom-up pathway is grounded in a systematic empirical review of proxemics-focused HRI studies, including both physical and simulated experiments, from which constructs and variables are derived directly from observed interaction behaviors.

By reconciling theory and AI-driven conceptual structures with empirically grounded evidence, the proposed taxonomy establishes a unified framework that connects abstract HRI models with measurable proxemic phenomena.

The resulting taxonomy systematically organizes proxemic dimensions into four interrelated clusters: Human, Robot, Environment, and Context. Together, these clusters capture the key dimensions shaping the proxemics spatial metrics (shape, measurement method, and morphing), including human activity and posture, robot design and behavior, environmental structure, task context, and the dynamic spatial properties of the proxemics metrics representations themselves, which are treated as proxemic output variables.

Our analysis revealed that existing HRI taxonomies (Onnasch and Roesler, 2021; Rodrigues et al., 2023; Singamaneni et al., 2024) do not fully capture the diversity of variables examined in proxemics-related studies. Several proxemics constructs were absent. Additionally, we introduce a distinctive Spatial metrics taxon that explicitly captures the geometric and dynamic properties of proxemic representations along with its variables (e.g., shape, measurement method, and morphing).

This more detailed treatment of proxemics highlights the need for a dedicated and systematic classification framework. Such a framework must explicitly consider robot characteristics and task attributes, human personal and physical dimensions, contextual and activity-related factors, and proxemics-specific variables, including spatial shape, distance measurements, and their potential for dynamic adaptation during interaction. This finding suggests that proxemics in HRI should be treated not merely as a fixed spatial parameter, but as a multidimensional and adaptive construct that reflects the dynamic interplay between human behavior, robot characteristics, environmental structure, and task context.

A unique contribution of this work is the incorporation of AI-based exploration as a complementary mechanism for advancing proxemics modeling in socially aware robot navigation. While traditional proxemics research relies on theory-driven formulations and controlled empirical studies, such approaches face inherent limitations when addressing the diversity, variability, and context-dependence of real-world social interactions. AI-based exploration enables the systematic discovery of proxemic patterns that may not be explicitly anticipated or easily formalized through conventional analytical methods. Rather than replacing theoretical or empirical foundations, this approach extends them by identifying latent structures and non-linear dependencies between proxemic variables. Through this process, the Spatial metrics emerged as an independent and essential taxon, providing a unique perspective and insights on the variables that define proxemics characteristics.

While this study proposes a taxonomy of proxemics, several limitations should be acknowledged. Our objective was not to elicit new opinions but to synthesize patterns across an existing interdisciplinary corpus. Therefore, clusters, categories, and variables were derived exclusively from the articles selected for the review; consequently, the taxonomy is constrained by the scope and potential biases of the existing literature.

The AI-assisted synthesis enabled systematic pattern exploration across a large volume of literature while preserving traceability to original sources. But, the conceptual integration, including the rationale for incorporating proxemics into the taxonomy, was determined by the authors through critical evaluation of the evidence base.

During the assembly of human-related taxa (Table 4), we observed variables discussed in theoretical papers but lacking empirical support regarding their effect on proxemics; for instance, culture, including regional and social norms (Eresha et al., 2013) or hand dominance and its influence on asymmetry (Gérin-Lajoie et al., 2008). Similarly, while compiling environment-related taxa (Table 5), we noted limited data for outdoor settings, with no representation in video-based studies.

Future research should explore additional variables that significantly influence proxemics in socially aware robot navigation. We acknowledge that alternative approaches, such as expert panels or focus groups, could generate additional perspectives, as highlighted by Francis et al. (2025), the field still exhibits notable gaps in robot design factors, participant diversity, environmental realism, task context, and the integration of multiple interacting variables.

Our suggestions below address gaps more specifically tied to the regulation of interpersonal distance and contextual variability. We frame these issues through a proxemic and interaction-centered taxonomy, complementing their algorithm-focused evaluation perspective.

- Robot design and behavior: While existing studies extensively cover robot design, there is limited exploration of specific robot physical size and behaviors that may influence proxemic zones.
- Participant diversity: Most studies involve primarily young people or students. Future research should incorporate a broader range of participants, including those of different ages, cultures, skills, and experience.
- Environmental context: Experiments are mostly conducted in controlled indoor spaces, such as laboratories or corridors, with only a few studies in outdoor or dynamic environments. Expanding experiments to diverse real-world settings is essential.
- Task context: Most studies examine robots moving from point to point without a purposeful context. Since context strongly affects HRI and proxemics, future research should integrate contextual activities into experimental designs.

Furthermore, research should advance beyond isolated examinations of individual variables by incorporating multiple variables simultaneously. Despite the added complexity this introduces into experimental design, such an approach would ensure more comprehensive and ecologically valid experimental contexts.

Finally, structuring these concepts as formal ontology represents a promising direction for future research. At the current stage of the field, many concepts remain heterogeneous in their definitions, operationalizations, and contexts of use. Our taxonomy provides a flexible and descriptive framework for organizing the existing knowledge base without imposing formal relationships that are not yet empirically established. Developing a formal ontology would require additional consensus on definitions and the relationships among variables.

## 7 Conclusion

With the growing deployment of autonomous service robots in shared environments, social navigation, and consequently proxemics has emerged as a critical challenge. For robots to be accepted in human-centered spaces, they must exhibit socially appropriate behaviors, as their actions directly influence humans’ sense of safety, comfort, and trust.

The proposed taxonomy of proxemics provides a systematic mapping and clustering of the variables that shape proxemic behavior in human-robot interaction. It captures the factors influencing both the shape and size of proxemic zones and organizes them within a structured framework. Unlike much of the previous literature, which often treats socially aware navigation as a set of isolated issues, this work conceptualizes proxemics as an asymmetric and dynamic construct that evolves during interaction with humans and the surrounding environment. It provides the output spatial metrics (shape, measurement method, and morphing) and structures the parameters influencing them.

As socially aware mobile robots become increasingly prevalent in real-world applications, integrating such adaptive proxemic models will be essential for guiding future developments in the field.

## Data Availability

The datasets analyzed in this study are available from the corresponding author upon request.
