# Supplementary material for: Advancing a taxonomy of proxemics for socially aware robot navigation
Source: Front Robot AI. 2026 May 20;13:1800762. doi: 10.3389/frobt.2026.1800762 (PMC13248410; doi:10.3389/frobt.2026.1800762)
Supplement: Supplementary file 1 [file Supplementaryfile1.pdf]

Advancing a Taxonomy of Proxemics for Socially Aware Robot Navigation

Supplementary material

Complete References list for Figure 2

FIGURE 2 – References by year

Year 2020

(Aghaei *et al.*, 2020; Aliasghari *et al.*, 2020; Clavero *et al.*, 2020; Gupta *et al.*, 2020; Kirks *et al.*, 2020; Lehmann, Rojik and Hoffmann, 2020; Leichtmann and Nitsch, 2020; Manso *et al.*, 2020; Miller *et al.*, 2020; Narayanan *et al.*, 2020; Patompak *et al.*, 2020; Rasouli and Tsotsos, 2020; Ruijten and Cuijpers, 2020; Siebert *et al.*, 2020)

Year 2021

(Belmonte *et al.*, 2021; Björn Petrak; Julia G. Stapels; Katharina Weitz; Friederike Eyssel; Elisabeth André, 2021; Bretin, Cross and Khamis, 2021; Calderita *et al.*, 2021; Camara and Fox, 2021b, 2021a; Daza *et al.*, 2021; Haarslev *et al.*, 2021; Hudspeth *et al.*, 2021; Liu, Liu and Gao, 2021; Mirsky *et al.*, 2021; Möller *et al.*, 2021; Ngo HQT, 2021; Webb, Mohamed and Lemaignan, 2021)

Year 2022

(Bellarbi *et al.*, 2022; Cathcart *et al.*, 2022; Galati *et al.*, 2022; Gallo D and Shreepriya Gonzalez-Jimenez, 2022; Gao and Huang, 2022; Ginés Clavero *et al.*, 2022; Holt *et al.*, 2022; Kobayashi *et al.*, 2022; Kunde *et al.*, 2022; Leichtmann *et al.*, 2022; Millán-Arias, Fernandes and Cruz, 2022; Neef *et al.*, 2022; Neggers *et al.*, 2022a, 2022b; Paez-Granados, Gupta and Billard, 2022; Pirk *et al.*, 2022; Rasouli *et al.*, 2022; Repiso, Garrell and Sanfeliu, 2022; Samarakoon *et al.*, 2022; Samarakoon, Muthugala and Jayasekara, 2022; Williamson *et al.*, 2022)

Year 2023

(Camara and Fox, 2023; Damholdt *et al.*, 2023; Davidson *et al.*, 2023; Fox and Loughlin, 2023; Francis *et al.*, 2023; Gallo *et al.*, 2023; Gasteiger, Hellou and Ahn, 2023; Georgiou *et al.*, 2023; Hanumantha, 2023; He *et al.*, 2023; Klüber and Onnasch, 2023; Leoste *et al.*, 2023; Mavrogiannis *et al.*, 2023; Moujahid *et al.*, 2023; Ribeiro and Moreno, 2023; Ribino, 2023; Singh *et al.*, 2023; Suresh *et al.*, 2023; Wang *et al.*, 2023; Xu *et al.*, 2023; Yamazoe *et al.*, 2023)

Year 2024

(Bilen *et al.*, 2024; Karwowski, Szynkiewicz and Niewiadomska-Szynkiewicz, 2024; Kim and Sung, 2024; Kim, Anthis and Sebo, 2024; Kim *et al.*, 2024; Leoste, Marmor and Heidmets, 2024; Mandal and Baraka, 2024; Marmor *et al.*, 2024; Masson *et al.*, 2024; Mende, Shneiderman and Boccanfuso, 2024; Neggers *et al.*, 2024; Nguyen and Ngo, 2024; Singamaneni *et al.*, 2024; Webb *et al.*, 2024; Yamanaka *et al.*, 2024)

Year 2025

(Apraiz *et al.*, 2025; Bretin, Cross and Khamis, 2025; Chen and Liu, 2025; Jung *et al.*, 2025; Mac *et al.*, 2025; Müller and Richert, 2025; Nahum, Edan and Oron-Gilad, 2025; Nolte *et al.*, 2025; Rajapakse, 2025; Ribeiro and Macharet, 2025; Song *et al.*, 2025; Spurný *et al.*, 2025)

REFERENCES

Aghaei, M. *et al.* (2020) “Single Image Human Proxemics Estimation for Visual Social Distancing.” Available at: http://arxiv.org/abs/2011.02018.

Aliasghari, P. *et al.* (2020) “Implementing a gaze control system on a social robot in multi-person interactions,” *SN Applied Sciences*, 2(6). Available at: https://doi.org/10.1007/s42452-020-2911-0.

Apraiz, A. *et al.* (2025) “Evaluating the Effect of Speed and Acceleration on Human Factors during an Assembly Task in Human–Robot Interaction (HRI),” *International Journal of Social Robotics*, 17(2), pp. 211–256. Available at: https://doi.org/10.1007/s12369-025-01224-7.

Bellarbi, A. *et al.* (2022) “A new approach for social navigation and interaction using a dynamic proxemia modeling,” *Evolutionary Intelligence*, 15(3), pp. 2207–2233. Available at: https://doi.org/10.1007/s12065-021-00633-7.

Belmonte, L.M. *et al.* (2021) “Feeling of safety and comfort towards a socially assistive unmanned aerial vehicle that monitors people in a virtual home,” *Sensors (Switzerland)*, 21(3), pp. 1–30. Available at: https://doi.org/10.3390/s21030908.

Bilen, B. *et al.* (2024) “Social Robot Navigation with Adaptive Proxemics Based on Emotions.” Available at: http://arxiv.org/abs/2401.17663.

Bretin, R., Cross, E. and Khamis, M. (2021) *Co-existing With Drones: A Virtual Exploration of Proxemic Behaviours and Users' Insights on Social Drones*.

Bretin, R., Cross, E. and Khamis, M. (2025) “Safety at Stake: How Individuals Task Prioritization Influences Human-Drone Proxemics,” *ACM Transactions on Human-Robot Interaction* [Preprint]. Available at: https://doi.org/10.1145/3748520.

Calderita, L. V. *et al.* (2021) “A new human-aware robot navigation framework based on time-dependent social interaction spaces: An application to assistive robots in caregiving centers[Formula presented],” *Robotics and Autonomous Systems*, 145. Available at: https://doi.org/10.1016/j.robot.2021.103873.

Camara, F. and Fox, C. (2021a) *Extending Quantitative Proxemics and Trust to HRI*. IEEE. (In Press. Available at: https://eprints.whiterose.ac.uk/.

Camara, F. and Fox, C. (2021b) “Space Invaders: Pedestrian Proxemic Utility Functions and Trust Zones for Autonomous Vehicle Interactions,” *International Journal of Social Robotics*, 13(8), pp. 1929–1949. Available at: https://doi.org/10.1007/s12369-020-00717-x.

Camara, F. and Fox, C. (2023) “A kinematic model generates non-circular human proxemics zones,” *Advanced Robotics* [Preprint]. Available at: https://doi.org/10.1080/01691864.2023.2263062.

Cathcart, C. *et al.* (2022) “Proactive Opinion-Driven Robot Navigation around Human Movers.” Available at: http://arxiv.org/abs/2210.01642.

Chen, X. and Liu, S. (2025) *Social-DSM: Design and Evaluation of Socially Aware Navigation Control through Extended Dynamical System Modulation*. Available at: https://ssrn.com/abstract=5251861.

Clavero, J.G. *et al.* (2020) “Defining Adaptive Proxemic Zones for Activity-aware Navigation.” Available at: http://arxiv.org/abs/2009.04770.

Damholdt, M.F. *et al.* (2023) “A Scoping Review of HRI Research on ‘Anthropomorphism’: Contributions to the Method Debate in HRI,” *International Journal of Social Robotics*. Springer Science and Business Media B.V., pp. 1203–1226. Available at: https://doi.org/10.1007/s12369-023-01014-z.

Davidson, M.J. *et al.* (2023) “Peripersonal tracking accuracy is limited by the speed and phase of locomotion.” Available at: https://doi.org/10.1101/2023.04.17.537137.

Daza, M. *et al.* (2021) “An approach of social navigation based on proxemics for crowded environments of humans and robots,” *Micromachines*, 12(2). Available at: https://doi.org/10.3390/mi12020193.

Fox, R. and Loughlin, O.' (2023) "People flow maps for socially conscious robot navigation." Available at: https://doi.org/10.13140/RG.2.2.19731.86560.

Francis, A. *et al.* (2023) "Principles and Guidelines for Evaluating Social Robot Navigation Algorithms." Available at: http://arxiv.org/abs/2306.16740.

Galati, G. *et al.* (2022) "Game theoretical trajectory planning enhances social acceptability of robots by humans," *Scientific Reports*, 12(1). Available at: https://doi.org/10.1038/s41598-022-25438-1.

Gallo, D. *et al.* (2023) "Investigating the Integration of Human-Like and Machine-Like Robot Behaviors in a Shared Elevator Scenario." Association for Computing Machinery (ACM), pp. 192–201. Available at: https://doi.org/10.1145/3568162.3576974.

Gallo D and Shreepriya Gonzalez-Jimenez (2022) *Exploring Machine-like Behaviors for Socially Acceptable Robot Navigation in Elevators*.

Gao, Y. and Huang, C.M. (2022) "Evaluation of Socially-Aware Robot Navigation," *Frontiers in Robotics and AI*. Frontiers Media S.A. Available at: https://doi.org/10.3389/frobt.2021.721317.

Gasteiger, N., Hellou, M. and Ahn, H.S. (2023) "Factors for Personalization and Localization to Optimize Human–Robot Interaction: A Literature Review," *International Journal of Social Robotics*, 15(4), pp. 689–701. Available at: https://doi.org/10.1007/s12369-021-00811-8.

Georgiou, N.C. *et al.* (2023) "Is Someone There or Is That the TV? Detecting Social Presence Using Sound," *ACM Transactions on Human-Robot Interaction*, 12(4). Available at: https://doi.org/10.1145/3611658.

Ginés Clavero, J. *et al.* (2022) "Impact of decision-making system in social navigation," *Multimedia Tools and Applications*, 81(3), pp. 3459–3481. Available at: https://doi.org/10.1007/s11042-021-11454-2.

Gupta, S. *et al.* (2020) "Corridor segmentation for automatic robot navigation in indoor environment using edge devices," *Computer Networks*, 178. Available at: https://doi.org/10.1016/j.comnet.2020.107374.

Haarslev, F. *et al.* (2021) "Context-aware social robot navigation," *Proceedings of the 18th International Conference on Informatics in Control, Automation and Robotics, ICINCO 2021*. SciTePress, pp. 426–433. Available at: https://doi.org/10.5220/0010554204260433.

Hanumantha, N. (2023) *IMPROVING THE AUTONOMOUS NAVIGATION OF A CARE ROBOT BY FOLLOWING SOCIAL NORMS IN A CARE ENVIRONMENT*.

He, K. *et al.* (2023) "Robot Gaze During Autonomous Navigation and Its Effect on Social Presence," *International Journal of Social Robotics* [Preprint]. Available at: https://doi.org/10.1007/s12369-023-01023-y.

Holt, D.J. *et al.* (2022) "Personal space increases during the COVID-19 pandemic in response to real and virtual humans," *Frontiers in Psychology*, 13. Available at: https://doi.org/10.3389/fpsyg.2022.952998.

Hudspeth, M. *et al.* (2021) "Effects of Interfaces on Human-Robot Trust: Specifying and Visualizing Physical Zones." Available at: http://arxiv.org/abs/2112.00779.

Jung, S. *et al.* (2025) "Proxemic Discomfort in Shared Spaces: The Role of Mobile Robot Behavior and Pedestrian State," *International Journal of Human-Computer Interaction* [Preprint]. Available at: https://doi.org/10.1080/10447318.2025.2526574.

Karwowski, J., Szynkiewicz, W. and Niewiadomska-Szynkiewicz, E. (2024) "Bridging Requirements, Planning, and Evaluation: A Review of Social Robot Navigation," *Sensors*, 24(9). Available at: https://doi.org/10.3390/s24092794.

Kim, I. and Sung, J. (2024) "New proxemics in new space: proxemics in VR," *Virtual Reality*, 28(2). Available at: https://doi.org/10.1007/s10055-024-00982-5.

Kim, S., Anthis, J.R. and Sebo, S. (2024) "A Taxonomy of Robot Autonomy for Human-Robot Interaction," *ACM/IEEE International Conference on Human-Robot Interaction*. IEEE Computer Society, pp. 381–393. Available at: https://doi.org/10.1145/3610977.3634993.

Kim, Y.S. *et al.* (2024) “Understanding human-robot proxemic norms in construction: How do humans navigate around robots?,” *Automation in Construction*, 164. Available at: https://doi.org/10.1016/j.autcon.2024.105455.

Kirks, T. *et al.* (2020) “Modelling proxemics for human-technology-interaction in decentralized social-robot-systems,” *Advances in Intelligent Systems and Computing*. Available at: https://doi.org/10.1007/978-3-030-39512-4_24.

Klüber, K. and Onnasch, L. (2023) “Keep your Distance! Assessing Proxemics to Virtual Robots by Caregivers.” Association for Computing Machinery (ACM), pp. 193–197. Available at: https://doi.org/10.1145/3568294.3580070.

Kobayashi, Y. *et al.* (2022) “Robot Navigation Based on Predicting of Human Interaction and its Reproducible Evaluation in a Densely Crowded Environment,” *International Journal of Social Robotics*, 14(2), pp. 373–387. Available at: https://doi.org/10.1007/s12369-021-00791-9.

Kunde, S. *et al.* (2022) *Let's run an online proxemics study! But, how do results compare to in-person?*

Lehmann, H., Rojik, A. and Hoffmann, M. (2020) “Should a small robot have a small personal space? Investigating personal spatial zones and proxemic behavior in human-robot interaction.” Available at: http://arxiv.org/abs/2009.01818.

Leichtmann, B. *et al.* (2022) “Personal Space in Human-Robot Interaction at Work: Effect of Room Size and Working Memory Load,” *ACM Transactions on Human-Robot Interaction*. Association for Computing Machinery. Available at: https://doi.org/10.1145/3536167.

Leichtmann, B. and Nitsch, V. (2020) “How much distance do humans keep toward robots? Literature review, meta-analysis, and theoretical considerations on personal space in human-robot interaction,” *Journal of Environmental Psychology*. Academic Press. Available at: https://doi.org/10.1016/j.jenvp.2019.101386.

Leoste, J. *et al.* (2023) “Keeping distance with a telepresence robot: A pilot study,” *Frontiers in Education*, 7. Available at: https://doi.org/10.3389/feduc.2022.1046461.

Leoste, J., Marmor, K. and Heidmets, M. (2024) “Nonverbal Behavior of Service Robots in Social Interactions-A Survey on Recent Studies.” Available at: https://doi.org/10.20944/preprints202401.1969.v1.

Liu, L., Liu, Y. and Gao, X.Z. (2021) “Impacts of human robot proxemics on human concentration-training games with humanoid robots,” *Healthcare (Switzerland)*, 9(7). Available at: https://doi.org/10.3390/healthcare9070894.

Mac, T.T. *et al.* (2025) “Human-Aware Robot Navigation Using Modified Social Force Model,” *Arabian Journal for Science and Engineering* [Preprint]. Available at: https://doi.org/10.1007/s13369-025-10383-8.

Mandal, A. and Baraka, K. (2024) “Using Proxemics as a Corrective Feedback Signal during Robot Navigation,” *Companion of the 2024 ACM/IEEE International Conference on Human-Robot Interaction*. New York, NY, USA: ACM, pp. 732–736. Available at: https://doi.org/10.1145/3610978.3640746.

Manso, L.J. *et al.* (2020) “Socnav1: A dataset to benchmark and learn social navigation conventions,” *Data*, 5(1). Available at: https://doi.org/10.3390/data5010007.

Marmor, K. *et al.* (2024) “Keeping social distance in a classroom while interacting via a telepresence robot: a pilot study,” *Frontiers in Neurorobotics*, 18. Available at: https://doi.org/10.3389/fnbot.2024.1339000.

Masson, M. *et al.* (2024) “ProxMetrics: modular proxemic similarity toolkit to generate domain-adaptable indicators from social media,” *Social Network Analysis and Mining*, 14(1). Available at: https://doi.org/10.1007/s13278-024-01282-1.

Mavrogiannis, C. *et al.* (2023) “Core Challenges of Social Robot Navigation: A Survey,” *ACM Transactions on Human-Robot Interaction* [Preprint]. Available at: https://doi.org/10.1145/3583741.

Mende, M., Shneiderman, B. and Boccanfuso, L. (2024) “Commentary: The Future of Human-Robot Interactions,” *Journal of Service Research* [Preprint]. Available at: https://doi.org/10.1177/10946705241296041.

Millán-Arias, C., Fernandes, B. and Cruz, F. (2022) “Proxemic behavior in navigation tasks using reinforcement learning,” *Neural Computing and Applications* [Preprint]. Available at: https://doi.org/10.1007/s00521-022-07628-0.

Miller, L. *et al.* (2020) “Come Closer: Experimental Investigation of Robots’ Appearance on Proximity, Affect and Trust in a Domestic Environment,” *Proceedings of the Human Factors and Ergonomics Society Annual Meeting*, 64(1), pp. 395–399. Available at: https://doi.org/10.1177/1071181320641089.

Mirsky, R. *et al.* (2021) “Conflict Avoidance in Social Navigation -- a Survey.” Available at: http://arxiv.org/abs/2106.12113.

Möller, R. *et al.* (2021) “A Survey on Human-aware Robot Navigation.” Available at: http://arxiv.org/abs/2106.11650.

Moujahid, M. *et al.* (2023) “Come Closer: The Effects of Robot Personality on Human Proxemics Behaviours.” Available at: http://arxiv.org/abs/2309.02979.

Müller, A. and Richert, A. (2025) “The Space Between Us: A Methodological Framework for Researching Bonding and Proxemics in Situated Group-Agent Interactions.” Available at: http://arxiv.org/abs/2506.11829.

Nahum, E., Edan, Y. and Oron-Gilad, T. (2025) “Investigating the Proxemics Shape in Social Navigation: An Exploratory User Study,” *Social Robotics: 16th International Conference, ICSR + AI 2024, Odense, Denmark, October 23–26, 2024, Proceedings,*. Springer, Singapore, pp. 168–177. Available at: https://doi.org/10.1007/978-981-96-3522-1_16.

Narayanan, V. *et al.* (2020) “ProxEmo: Gait-based Emotion Learning and Multi-view Proxemic Fusion for Socially-Aware Robot Navigation.” Available at: http://arxiv.org/abs/2003.01062.

Neef, N.E. *et al.* (2022) “What is Appropriate? On the Assessment of Human-Robot Proxemics for Casual Encounters in Closed Environments.” Available at: https://doi.org/10.21203/rs.3.rs-2329385/v1.

Neggers, M.M.E. *et al.* (2022a) “Determining Shape and Size of Personal Space of a Human when Passed by a Robot,” *International Journal of Social Robotics*, 14(2), pp. 561–572. Available at: https://doi.org/10.1007/s12369-021-00805-6.

Neggers, M.M.E. *et al.* (2022b) “The effect of robot speed on comfortable passing distances,” *Frontiers in Robotics and AI*, 9. Available at: https://doi.org/10.3389/frobt.2022.915972.

Neggers, M.M.E. *et al.* (2024) “Comfortable Crossing Strategies for Robots,” *International Journal of Social Robotics* [Preprint]. Available at: https://doi.org/10.1007/s12369-024-01127-z.

Ngo HQT (2021) “Recent researches on human-aware navigation.”

Nguyen, T.T. and Ngo, T.D. (2024) “Spatiotemporal Motion Profiles for Cost-Based Optimal Approaching Pose Estimation,” *2024 IEEE/SICE International Symposium on System Integration, SII 2024*. Institute of Electrical and Electronics Engineers Inc., pp. 92–98. Available at: https://doi.org/10.1109/SII58957.2024.10417396.

Nolte, D. *et al.* (2025) “Investigating proxemics behaviors towards individuals, pairs, and groups in virtual reality,” *Virtual Reality*, 29(2). Available at: https://doi.org/10.1007/s10055-025-01127-y.

Paez-Granados, D., Gupta, V. and Billard, A. (2022) “Unfreezing Social Navigation: Dynamical Systems based Compliance for Contact Control in Robot Navigation.” Available at: https://doi.org/10.1109/ICRA46639.2022.9811772.

Patompak, P. *et al.* (2020) “Learning Proxemics for Personalized Human–Robot Social Interaction,” *International Journal of Social Robotics*, 12(1), pp. 267–280. Available at: https://doi.org/10.1007/s12369-019-00560-9.

Petrak Björn; Julia G. Stapels; Katharina Weitz; Friederike Eyssel; Elisabeth André (2021) “To move or not to move Social acceptability of robot proxemics behavior depending on user emotion.”

Pirk, S. *et al.* (2022) “A Protocol for Validating Social Navigation Policies.” Available at: http://arxiv.org/abs/2204.05443.

Rajapakse, J. (2025) *A Study on Multi-Perspective Switching Interaction and Passive Camera Calibration in Virtual Reality Based on Proxemics. Exploring User-Object Proxemics in VR Game “Feather Forest.”*

Rasouli, A. *et al.* (2022) *Unfreezing autonomous vehicles with game theory, proxemics, and trust*.

Rasouli, A. and Tsotsos, J.K. (2020) “Autonomous vehicles that interact with pedestrians: A survey of theory and practice,” *IEEE Transactions on Intelligent Transportation Systems*. Institute of Electrical and Electronics Engineers Inc., pp. 900–918. Available at: https://doi.org/10.1109/TITS.2019.2901817.

Repiso, E., Garrell, A. and Sanfeliu, A. (2022) “Adaptive Social Planner to Accompany People in Real-Life Dynamic Environments,” *International Journal of Social Robotics* [Preprint]. Available at: https://doi.org/10.1007/s12369-022-00937-3.

Ribeiro, C.C.G. and Macharet, D.G. (2025) “Beyond the Plane: A 3D Representation of Human Personal Space for Socially-Aware Robotics.” Available at: http://arxiv.org/abs/2506.13937.

Ribeiro, R. and Moreno, P. (2023) “Socially reactive navigation models for mobile robots in dynamic environments.” Available at: http://arxiv.org/abs/2310.09916.

Ribino, P. (2023) “The role of politeness in human–machine interactions: a systematic literature review and future perspectives,” *Artificial Intelligence Review*, 56. Available at: https://doi.org/10.1007/s10462-023-10540-1.

Ruijten, P.A.M. and Cuijpers, R.H. (2020) “Do not let the robot get too close: Investigating the shape and size of shared interaction space for two people in a conversation,” *Information (Switzerland)*, 11(3). Available at: https://doi.org/10.3390/info11030147.

Samarakoon, S.M.B.P. *et al.* (2022) “Adapting approaching proxemics of a service robot based on physical user behavior and user feedback,” *User Modeling and User-Adapted Interaction* [Preprint]. Available at: https://doi.org/10.1007/s11257-022-09329-8.

Samarakoon, S.M.B.P., Muthugala, M.A.V.J. and Jayasekara, A.G.B.P. (2022) “A Review on Human–Robot Proxemics,” *Electronics (Switzerland)*. MDPI. Available at: https://doi.org/10.3390/electronics11162490.

Siebert, F.W. *et al.* (2020) “The Influence of Distance and Lateral Offset of Follow Me Robots on User Perception,” *Frontiers in Robotics and AI*, 7. Available at: https://doi.org/10.3389/frobt.2020.00074.

Singamaneni, P.T. *et al.* (2024) “A survey on socially aware robot navigation: Taxonomy and future challenges,” *The International Journal of Robotics Research* [Preprint]. Available at: https://doi.org/10.1177/02783649241230562.

Singh, K.J. *et al.* (2023) “Behavior of Delivery Robot in Human-Robot Collaborative Spaces During Navigation,” *Intelligent Automation and Soft Computing*, 35(1), pp. 795–810. Available at: https://doi.org/10.32604/iasc.2023.025177.

Song, H. *et al.* (2025) “No Need for Speed? The Impact of Delivery Robot Speed on Passersby’s Perceived Comfort and Safety and Preferred Signaling Distance,” *International Journal of Social Robotics*, 17(7), pp. 1277–1288. Available at: https://doi.org/10.1007/s12369-025-01269-8.

Spurný, T. *et al.* (2025) “Dynamic Proxemic Model for Human–Robot Interactions Using the Golden Ratio,” *Applied Sciences*, 15(15), p. 8130. Available at: https://doi.org/10.3390/app15158130.

Suresh, A. *et al.* (2023) “Robot Navigation in Risky, Crowded Environments: Understanding Human Preferences.” Available at: http://arxiv.org/abs/2303.08284.

Wang, Z. *et al.* (2023) “The Effects of Natural Sounds and Proxemic Distances on the Perception of a Noisy Domestic Flying Robot,” *ACM Transactions on Human-Robot Interaction* [Preprint]. Available at: https://doi.org/10.1145/3579859.

Webb, N. *et al.* (2024) “Co-Movement and Trust Development in Human-Robot Teams.” Available at: http://arxiv.org/abs/2409.20218.

Webb, N., Mohamed, Y. and Lemaignan, S. (2021) “Measuring Visual Social Engagement from Proxemics and Gaze in the Real World,” *ACM/IEEE International Conference on Human-Robot Interaction*. IEEE Computer Society, pp. 552–554. Available at: https://doi.org/10.1145/3434074.3447233.

Williamson, J.R. *et al.* (2022) “Digital Proxemics: Designing Social and Collaborative Interaction in Virtual Environments,” *Conference on Human Factors in Computing Systems - Proceedings*. Association for Computing Machinery. Available at: https://doi.org/10.1145/3491102.3517594.

Xu, X. *et al.* (2023) “Understanding Dynamic Human-Robot Proxemics in the Case of Four-Legged Canine-Inspired Robots.” Available at: http://arxiv.org/abs/2302.10729.

Yamanaka, R. *et al.* (2024) “Characteristics of Personal Space during Human-Robot Interactions: A Systematic Review,” *Journal of Computer and Communications*, 12(05), pp. 107–123. Available at: https://doi.org/10.4236/jcc.2024.125008.

Yamazoe, H. *et al.* (2023) “Analysis of impressions of robot by changing its motion and trajectory parameters for designing parameterized behaviors of home-service robots,” *Intelligent Service Robotics*, 16(1), pp. 3–18. Available at: https://doi.org/10.1007/s11370-022-00447-1.
